# Does stem profile have an impact on the failure patterns in revision total knee arthroplasty?

**DOI:** 10.1007/s00402-022-04683-z

**Published:** 2022-11-30

**Authors:** Alexander Maslaris, Eleftherios Tsiridis, Carsten Schoeneberg, Bastian Pass, Georgios Spyrou, Alexandros Maris, Georg Matziolis

**Affiliations:** 1 Department of Orthopedics, Waldkliniken Eisenberg, Jena University Hospital, Klosterlausnitzer Str. 81, 07607 Eisenberg, Germany; 2 Department of Orthopedics and Trauma Surgery, Alfried-Krupp Hospital Essen, Alfried-Krupp-Straße 21, 45131 Essen, Germany; 3 Academic Orthopedic Department, Papageorgiou General Hospital, Aristotle University Medical School, Thessaloniki, GRC Greece; 4 Department of Orthopedics, Great Ormond Street Hospital NHS Foundation Trust, Great Ormond Street, London, WC1N 3JH UK

**Keywords:** Stem profile, Stem design, Conical stems, Cemented fixation, Revision total knee arthroplasty, Failure pattern, Aseptic loosening

## Abstract

**Introduction:**

Revision total knee arthroplasty (RTKA) has been increasing continuously. The results of RTKA still remain unsatisfactory. Failure patterns and risk factors in RTKA were thoroughly analyzed, with periprosthetic joint infections (PJI) and aseptic loosening remaining at the forefront of re-revision (ReRTKA) causes. While there is evidence that stem profile impacts the revisability of cemented implants, its association with the modes of RTKA failure is unknown.

**Methods:**

50 consecutive ReRTKA performed in a single orthopedic center during 2016–2017 were retrospectively analyzed. The cases were stratified according to age, sex, number of preexisting revisions, fixation technique, stem design and causes of re-revision. All explanted implants with conical vs. cylindrical stem profiles were compared.

**Results:**

Mean age was 67 ± 11.5, and 54% were females. 72% of the cases had ≥ 3 previous revisions. 88% were full-cemented, 3% hybrid and 9% press-fit stems. 36% of the RTKA had conical, 58% cylindrical and 6% combined stem profiles. 92% of the RTKA components were removed. Removal causes were: PJI (52.2%), aseptic loosening (34.8%), implant malposition (9.8%), painful knee (1.1%) and instability (2.2%). While the overall RTKA failure patterns were equally distributed between conical and cylindrical stems, subgroup analysis of only cemented ReRTKA revealed a higher incidence of aseptic loosening within cylindrical stem profiles (46.7% vs. 25.7%, *P* = 0.05).

**Conclusion:**

Stem profile may have an impact on the process of aseptic loosening in cemented non-metaphyseal engaging RTKA, with cylindrical designs tending to worse outcomes than conical designs. Large cohort studies could provide more clarity on current observation.

## Introduction

Data from national arthroplasty registries and the current available literature indicate an increasing incidence of revision total knee arthroplasty (RTKA) and with it of re-revisions (ReRTKA) worldwide [1–9]. This trend poses a great challenge for the present and an even greater burden for the future healthcare and economic system [10–12].

With RTKA failure rates ranging between 10–30%, the RTKA outcomes are still unsatisfactory [13–24]. The two most common causes of ReRTKA remain the periprosthetic joint infection (PJI) (21–46%), followed by aseptic loosening (20–30.5%) [14, 15, 19, 20, 24, 25]. Thus, the importance of identifying and preventing potential risk factors of RTKA failure becomes even greater.

Stem design seems to play an important role not only in the primary stability of RTKA implants [26], but also in the revisability of ReRTKA and, therefore, in their overall outcomes. Conical stem profile is a macroscopic design property defined by its angle and proportion of the conical part within a stem length (Table 1). Conical stem designs are associated with a significantly easier removal than cylindrical stems in the setting of well-fixed cemented RTKA stem extensions [27, 28]. As far as our experience goes, we can only confirm this observation, as increased morbidity is often linked to revisions of well-fixed cylindrical long stems, where often an extended osteotomy is necessary to facilitate controllable component removal. Although existing data from the domain of revision total hip arthroplasty suggest that conical stems are associated with better implant osteointegration rates than cylindrical stems in complex revision cases [29], no literature exists so far, in terms of evaluating the impact of conical stem profile on the failure patterns in revision total knee arthroplasty implants.

**Table 1:** Explanted RTKA implants and the conical profile of their stem extensions, as measured using the preoperative planning software MediCAD

RTKA model (manufacturer)	No	Stem design	Conical angle	Conical proportion
MUTARS^®^ GenuX^®^ MK (Implantcast, Buxtehude, Germany)	4	Cy (Co)	0° (8°)	30% proximal
Endo-Model RH prosthesis (Waldemar LINK, Hamburg, Germany)	17	Co	2°	100%
Zimmer^®^ NexGen^®^ LCCK (ZIMMER BIOMET, Warsaw, USA)	10	Cy	0°	0%
Zimmer^®^ NexGen^®^ RHK (ZIMMER BIOMET, Warsaw, USA)		Cy	0°	0%
P.F.C.™ SIGMA TC3 (DePuy Synthes Joint Reconstruction, Warsaw, USA)	6	Cy (Co)*	0° (1.6°–2.7°)	0% (100%)
LCS COMPLETE™ RKS (DePuy Synthes Joint Reconstruction, Warsaw, USA)	2	Cy (Co)*	0° (1.6°–2.7°)	0% (100%)
M.B.T. Revision Tray Monobloc (DePuy Synthes Joint Reconstruction, Warsaw, USA)	1	Co*	8°	100%
LEGION^®^ RK or HK (Smith & Nephew, Inc., Memphis, USA)	3	Cy	0°	0%
RT-PLUS Modular Knee (Smith & Nephew, Inc., Memphis, USA)	4	Cy*	0°	0%
EndoRo^®^ AS KRS (B.Braun, Aesculap AG, Tuttlingen, Germany)	3	Cy (Co)	0° (2–3°)	23% distal

*Depending on the length and fixation technique, some otherwise cylindrical stems may also possess conical profiles [e.g., the cemented tapered universal stems with length 90–150 mm and conical angles 1.6°–2.7° (DePuy)]. Furthermore, while the RT-PLUS modular knee possesses a cylindrical stem, the cemented RT-PLUS monobloc has a conical stem design (95 mm/4.5°–18°). Other RTKA implants may have different stem profiles within the same model depending on the side of the joint, usually with cylindrical designs on the femoral side and conical designs on the tibial side [e.g., the M.B.T. Revision Tibia Tray (DePuy) with a monobloc conical stem (61.8 mm/8°), and the Natural Knee II Revision (Zimmer) with cylindrical femoral stems and conical tibial stems (90–215 mm/12°–5.1°)]

CAVE: measurements may vary from the original manufacturer’s values

The purpose of the current study was to perform an observational analysis of the RTKA failure modes that have led to ReRTKA and compare the causes of implant removal between conical and cylindrical stem profiles. Relevant factors such as anchoring technique of the stem (full-cemented, press-fit, hybrid), metaphyseal fixation (cones/sleeves) and the case complexity (number of previous revisions) were taken into account. Lastly, a thorough review of the exiting literature on the outcomes of stemmed RTKA implants after (a) complex primary and (b) revision cases was conducted in “Discussion” to serve as benchmark for our findings and to facilitate the understanding of its background.

## Methods

Ethical approval from the local Institutional Review Board was granted. A single-center retrospective epidemiological analysis was initiated in an orthopedic teaching hospital.

All consecutive ReRTKA with implant removal that took place between 2016 and 2017 including constrained and semi-constrained implant revisions were recruited. Demographic data were collected using the institutional database. All RTKA failure patterns requiring implant removal were identified.

Exclusion criteria were re-revisions with only spacer exchange/removal or liner exchange, isolated patella revisions, explantation of implants that did not possess a stem extension or revisions due to malignancies. Lastly, RTKA reimplantations in the context of a two-stage exchange were also not considered in this study.

**Fig. 1:**
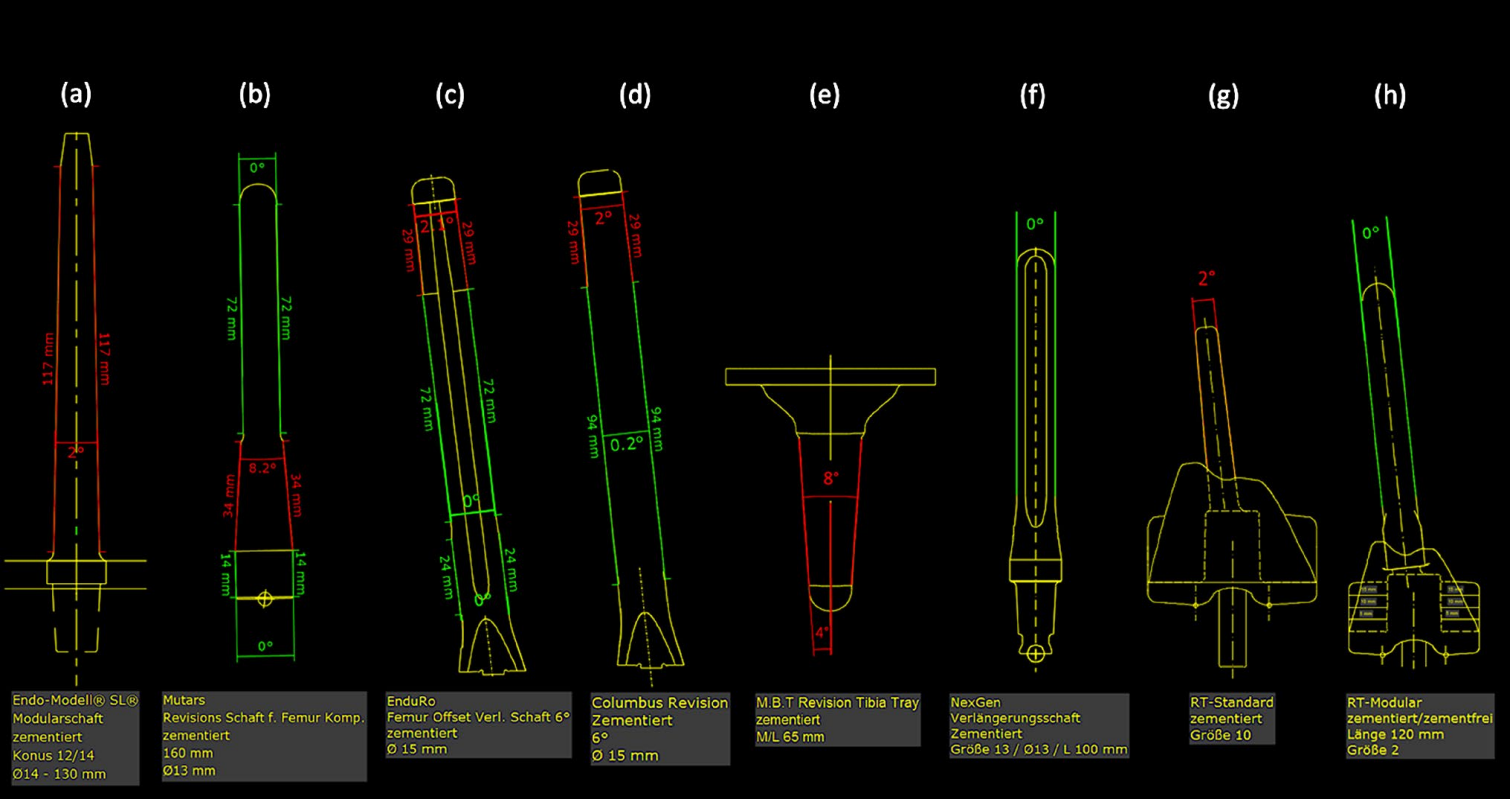
**a–h** Measurements of the conical stem profile of some established RTKA systems using the preoperative planning software MediCAD® orthopedic, German version. Red color: conical part of the stem, green color: cylindrical part of the stem. **a** ENDO-Model SL cemented (LINK), **b** MUTARS GenuX cemented (Implantcast), **c** Enduro cemented (Aesculap), **d** Columbus cemented (Aesculap), **e** M.B.T. Revision Tibia Tray cemented (DePuy), **f** NexGen LCCK cemented (Zimmer), **g** RT-PLUS Monobloc cemented (Smith & Nephew), **h** RT-PLUS Modular cemented and cementless (Smith & Nephew). CAVE: Measurements may vary from the original manufacturer’s values

All RTKA stems were stratified according to their profile in (1) conical (*Co*), (2) cylindrical (*Cy*) and (3) conical–cylindrical or cylindrical–conical combined designs (*Combi)*. The angles of the conical stem profile were measured using the 2D preoperative planning software MediCAD® orthopedic solution (MediCAD Hectec GmbH, Altdorf/Landshut, Germany) (Fig. 1a–h).

The fixation techniques of the stems were divided into (1) full-cemented, (2) cementless and (3) hybrid. The numbers of previous RTKA performed in each case until index surgery were noticed and quantified in: one previous (1R), two previous (2R), three previous (3R) and more than three previous revisions (> 3R). The removed femoral and tibial components including their corresponding stem extensions and the presence or not of a metaphyseal fixation were separately analyzed.

The obtained data were then compared between RTKA implants with (1) conical stems and (2) cylindrical stems. A subgroup analysis including only full-cemented stems was also conducted to improve sample homogeneity.

The software IBM SPSS Statistics (Version 25) was used to carry out statistical analyses. For differences between groups, the non-parametric two-sided Mann Whitney *U*-test or Kruskal Wallis test were performed. The Chi-Square test was used to compare frequencies.

## Results

A total of 50 ReRTKA cases (100 components, respectively) with stem revision were included in the study. All demographics and epidemiological data collected are summarized as follows (Table 2). The mean age of the patients was 67 ± 11.5 years and 54% were females. From the revised RTKA implants, 18 (36%) had conical stems, 29 (58%) cylindrical stems and 3 (6%) combined stem designs. 88% of the stems revised were full cemented and 12% cementless or hybrid (only metaphysis cemented). 72% of the cases were complex ReRTKA with three or more previous revisions.

**Table 2:** Demographics and epidemiological data of all ReRTKA included in the study, divided into *Co*, *Cy* and *Combi* stem profiles

	Conical *(Co)*	Cylindrical *(Cy)*	Combination *(Combi)*	All	Sign *Co vs. Cy*
Age	67.7 ± 9.7	67.9 ± 9.1	58 ± 27.5	67 ± 11.5	*0.99*
Gender (females)	12	13	2	27	*0.84*
Knees components (*f & t*)	18	29	3	50	0.02*
	36	58	6	100	
Component fixation					
*Cemented*	36	50	2	88	*0.49*
*Press fit*	0	6	3	9	0.05*
*Hybrid*	0	2	1	3	*0.26*
Component removals					
***All***	**35**	**52**	**5**	**92**	*0.71*
*f*	17	28	2	47	*0.73*
*t*	18	24	3	45	*0.72*
Causes of removed component					
*PJI*	20	26	2	48	*0.65*
*Aseptic loosening*	9	23	0	32	*0.16*
*Malposition*	4	3	2	9	*0.36*
*Painful knee*	0	0	1	1	
*Instability*	2	0	0	2	*0.08*
Previous RTKAs					
*1R*	2	6	0	8	*0.43*
*2R*	2	3	1	6	*0.93*
*3R*	5	11	2	18	*0.56*
> *3R*	9	9	0	18	*0.30*

*Co*  conical stem design, *Cy*  cylindrical stem design, *Combi*  combined stem designs, *RTKA*  revision total knee arthroplasty, *PJI*   periprosthetic joint infection, *1R*  one previous revision, *2R*  two previous revisions, *3R*  three previous revisions,  > *3R*  more than three previous revisions, *1 s*  one-stage exchange RTKA, *2 s*  two-stage exchange RTKA, *f*  femoral, *t*  tibial

*Differences are significant at the 0.05 level

A total of 92 of the 100 components included in the study (92%) were explanted, 51% of which were on the femoral side and 49% on the tibial side of the joint. Within all removed components, there was 1 (1.1%) metaphyseal cone on the femoral side and 12 (13%) sleeves (6 femoral, 6 tibial), all of which were combined with cementless/hybrid stem fixation. 35 out of 36 (97.2%) conical stems vs. 52 out of 58 (89.7%) cylindrical stems and 5 out of 6 (83.3%) combined design stems were removed during index ReRTKA.

The comparative analysis between conical and cylindrical stem revisions showed a good sample homogeneity with no statistical differences in revision complexity and the other demographic and epidemiological parameters included in the study.

The RTKA failure patterns of all 92 removed components in this cohort, sorted in descending order, were as follows: 52.2% PJI (47.8% on well-fixed implants and 4.4% on loose implants), 34.8% aseptic loosening, 9.8% implant malposition, 1.1% painful knee and 2.2% instability. Incidence comparison of the ReRTKA causes that led to implant removal between the two groups (*Co* vs. *Cy* stems) revealed an equal distribution of all RTKA failure patterns between the two stem designs with no statistical differences.

**Fig. 2:**
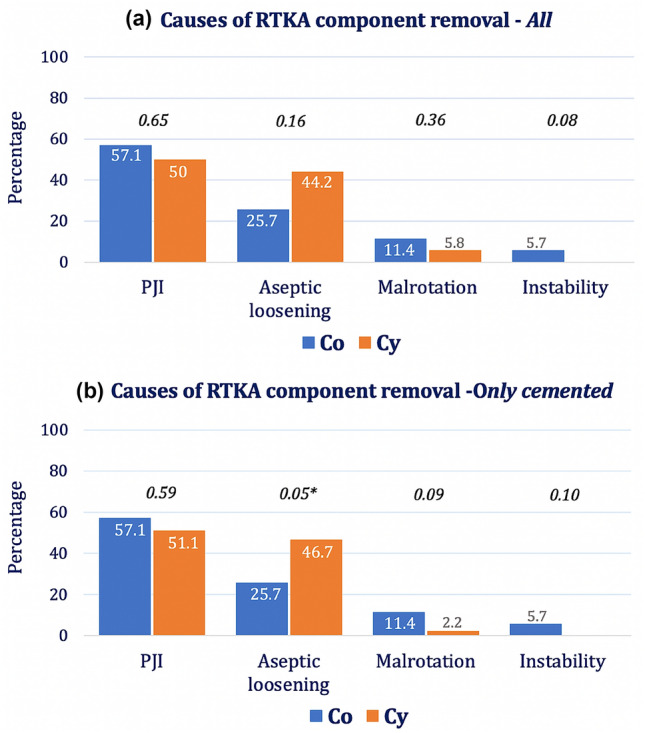
**a**, **b** Comparison of failure patterns of the RTKA components removed between Co and Cy stem profiles for **a** all RTKA implants included in the study (cemented and press-fit stems) and **b** only cemented stems. The values above the bars represent the level of significance as assessed by the *Chi square test*. *Significance at the 0.05 level

After extracting all cementless and hybrid stem fixation techniques and implants with metaphyseal fixations (cone/sleeves), to improve sample homogeneity, a subgroup analysis involving only cemented stems was conducted (Table 3). All conical stems (36/36) vs. 86.2% (50/58) of cylindrical stems were cemented. 35 out of 36 (97.2%) cemented conical stems vs. 45 out of 50 (90%) cemented cylindrical stems were explanted during index ReRTKA. The RTKA failure patterns of all 82 removed cemented components were as follows: 54.9% PJI, 36.6% aseptic loosening, 6.1% implant malposition, 0% painful knee and 2.4% instability. While there were no statistical differences in the incidences of PJI, malposition and instability between conical and cylindrical stem revisions, aseptic loosening occurred significantly more often in the full-cemented cylindrical stems (*P* = 0.05) when compared with conical stem profiles (Fig. 2).

**Table 3:** Subgroup analysis of the RTKA failure patterns including only full-cemented RTKA stems, divided into *Co*, *Cy* and *Combi* stem profiles

	Conical *(Co)*	Cylindrical *(Cy)*	Combination *(Combi)*	All	Sign *Co vs. Cy*
All components	**36**	**58**	**6**	**100**	
All cemented components	36	50	2	88	*0.02*
Removed cemented components					
***All***	**35**	**45**	**2**	**82**	*0.19*
*f*	17	23	1	41	
*t*	18	22	1	41	
Causes of removed cemented implants					
*PJI*	20	23	2	45	*0.59*
*Aseptic loosening*	9	21	0	30	0.05*
*Malposition*	4	1	0	5	*0.09*
*Instability*	2	0	0	2	*0.10*

*Co* conical stem design, *Cy*  cylindrical stem design, *Combi*  combined stem designs, *RTKA*  revision total knee arthroplasty, *Re-RTKA*  Re-revision, *PJI*  periprosthetic joint infection, *f*  femoral, *t*  tibial

*Differences are significant at the 0.05 level

## Discussion

### Main findings of the study

The main finding of this study is the association found between aseptic loosening and stem profile in failed cemented RTKA implants. While there was an equal distribution of all RTKA failure patterns observed between conical and cylindrical stem profiles in our initial cohort of 50 ReRTKA cases, with PJI remaining the most common failure reason, followed by aseptic loosening, a subgroup analysis of failed RTKA components with only full-cemented stems revealed a higher incidence of aseptic loosening in implants with cylindrical stems when compared to those with conical stem profiles reaching statistical significance (46.7% vs. 25.7%, *P* = 0.05).

### The role of stem in RTKA implant fixation

The role of stems in the primary and secondary stability of constrained implants is well known. It supports implant fixation by improving the distribution of shear loads, especially when epi-/metaphyseal bony defects > 5 mm are involved. It reduces the stresses on the bone–cement interface [30–32] and on the stem–condyle junction, which may otherwise lead to trunnion failure especially when no additional metaphyseal support coexists. Thus, based on the concept of the three fixation zones in RTKA [33], the use of cones [34, 35] or sleeves [36–38] in combination with a stem extension is becoming even more popular, as it shows improved clinical outcomes and lower rates of aseptic loosening [39–41]. Furthermore, offset stems can be useful in preventing component impingement and malalignment, as seen with straight diaphyseal-engaging cementless stems on bones with deformities [42].

Cement fixation in RTKA has various advantages [21, 32, 43–47]. It compensates incongruences of the cancellous bone and increases the contact surface to the implant, providing a more homogeneous load distribution [48]. It is for that reason, and at same time due to the excellent long-term results, that many surgeons traditionally advocate cemented stem fixation [49–54]. Nevertheless, there are also some drawbacks and pitfalls to consider, such as that of a progressive resorption below the tibial component that may occasionally occur, or the challenging revision of cemented voluminous cylindrical stems that are still well-fixed during their removal. Previous studies pointed out that short cemented stems with a conical profile angle of at least 0.5° may be for this reason preferable [27, 28, 55].

The mechanical properties of a stem design in RTKA involves several features, each one of them playing a decisive role in the biomechanics and anchoring mechanism of RTKA implants.

1. *Surface roughness*
  Depending on the values of implant surface roughness, cement adhesion and abrasion properties can differ [56, 57]. Smoothly polished stems are most commonly used in cemented fixation. Although they can depict micromotions within the intact cement sheath, no damage of interface occurs. On the other hand, stems with a rough surface provide a stronger bond with the cement; however, a microscopic undercutting that takes place within the cement–implant interface may lead to an earlier stem migration and, inevitably, to failure of the cement integrity and implant loosening. The cutoff values of implant surface roughness, above which a significant increase of loosening risk may occur, is yet undefined and difficult to examine. Thus, various satin to matte manufactured stem extensions are commonly used for both, cemented and cementless fixation techniques.
2. *Cross-sectional design*
  The cross-sectional design of cemented tibial short stems in primary TKA appears to have an impact on the surrounding bone density. In a prospective study of 20 cemented TKA, cylindrical stems revealed in a follow-up (FU) of 7 years more heterogeneous BMD changes beneath the tibial component compared to cruciform stems. The most density decrease was observed on the medial side. This was postulated as a risk for component migration [58]. Stem extensions with flutes and pronounced corners are commonly used in press-fit fixations to engage cortical bone and provide a higher resistance against torsional loads equal to those observed in daily activities [59, 60]. In cases of cemented fixation, the pronounced corners produce higher peak stresses on the surrounding cement, which may, subsequently, increase the risk of breakage of the cement mantle and lead to early loosening. Thus, rounded and scalloped stem designs have become the standard [61].
3. *Size (length and diameter)*
  Lee et al. [62] examined 65 press-fit semi-constrained knee prostheses, the impact of stem design (length and diameter) and the canal filling ratio (CFR) on the incidence of aseptic loosening. In an FU of 24 months, the incidence rates of aseptic loosening were equal for the femoral and tibial components. A CFR of > 0.85 was associated with reduced rates of aseptic loosening. They suggested either a CFR > 0.85 or a > 0.70 with > 4.3 cm stem engagement, tibial, and > 2 cm stem engagement, femoral. While, in hybrid or cementless fixation techniques maximal canal filling of press-fit stems is advocated [63], biomechanical studies have shown that equal primary stability can be achieved also via metaphyseal cemented stems [64]. However, according to recent studies on cemented short stems, a femoral canal wider than 19 mm inner diameter at 20 cm proximal to the joint line was linked to higher rates of aseptic loosening after cemented rotating hinge RTKA implants [39, 65, 66]. Finally, well-fixed cemented voluminous cylindrical stems are associated with an increased index of surgical invasiveness when revised und should be possibly avoided, especially if a potential re-revision is highly suspected (e.g. increased risk for infection recurrence) [28, 55].
4. *Conical stem profile*
  According to recent data, cemented conical stems can be more easily removed than cylindrical shaft extensions and, therefore, may be associated with reduced invasiveness and surgery time during exchange procedures [27, 28].

**Fig. 3:**
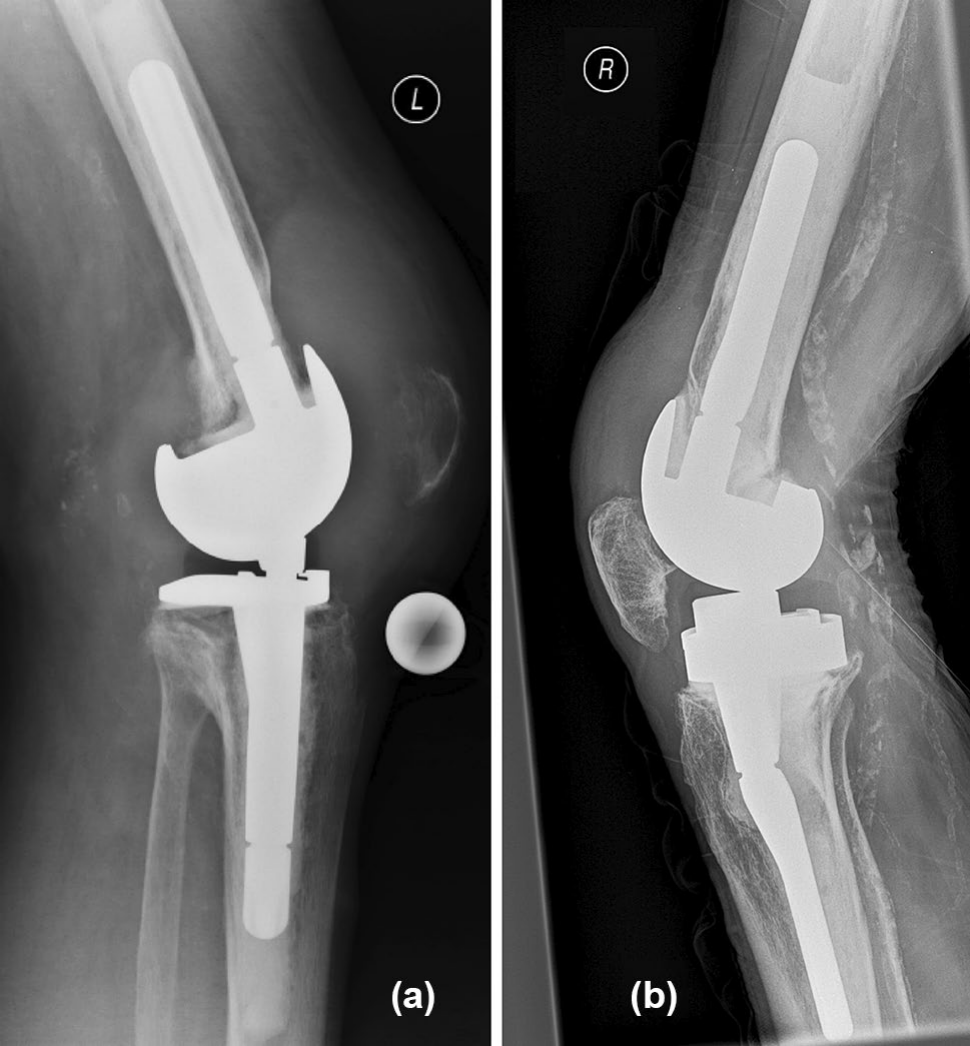
**a**, **b** Two representative cases of RTKA implants with well-fixed cemented cylindrical femoral stems from an external hospital, which were introduced at our emergencies due to PJI

Regarding stem fixation mechanism, cylindrical press-fit stems require a shorter engagement length than conical stems to achieve primary stability [26]. Nevertheless, stem engagement of less than 4 cm is associated with a higher risk of implant loosening [54, 67] and shaft pain due to peak stresses at the tip of the stem may result. Thus, tightly implanted diaphyseal press-fit stems can lead to end of stem pain and poor patient satisfaction [32, 43–47]. In a recent retrospective study of 20 cemented TKA, cylindrical stems revealed heterogeneous epiphyseal BMD decreases, which was postulated as a risk factor for component migration [58]. While cylindrical stems are commonly used with press-fit or hybrid fixation techniques and short conical stems with cemented fixation methods, it is not infrequent that orthopedic surgeons are confronted with revisions of cemented cylindrical stems or stem designs that possess in most parts of stem length a cylindrical profile (Fig. 3a, b).

### The role of stem design in RTKA outcomes: a comparative review of the literature

Despite the existing literature upon stem design in RTKA so far, there is still not much evidence dealing with the impact of stem profile on failure patterns of RTKA and its association with aseptic loosening.

**Fig. 4:**
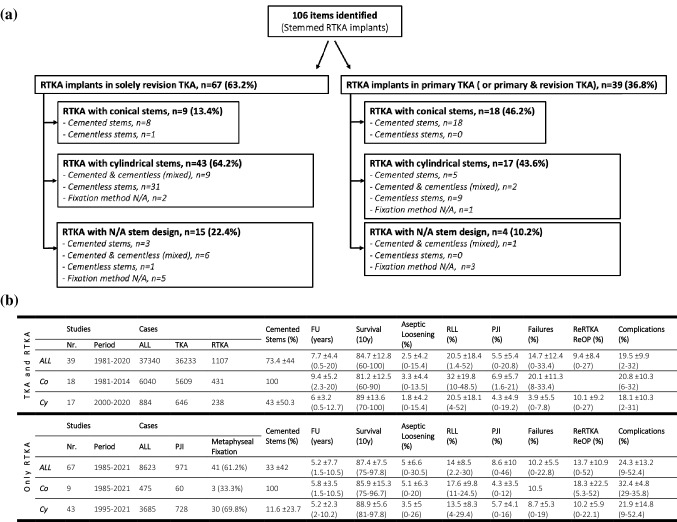
**a**, **b** Literature analysis: **a** process flowchart of the included studies for (left) solely revisions and (right) primary TKA (± revisions), divided according to stem profile (conical vs. cylindrical) and fixation technique of the stem (cemented or cementless). **b** Summary of overall outcomes separating TKA from RTKA, and Co from Cy stem profiles. Use of cement on stems and metaphyseal fixation (cones/sleeves) are noted in percentage

A comprehensive overview of the literature to assess survival rates and incidences of aseptic loosening and PJI with particular focus on the conical profile of RTKA stems (Co vs. Cy designs) was conducted to serve as benchmark and to facilitate a better understanding of our observations. A total of 106 studies including both, solely revisions (*n* = 67) (Table 4) and complex primary TKA cases (± revisions) (*n* = 39) (Table 5), have been reviewed and filtered as shown in the flowchart (Fig. 4a, b).

**Table 4:** Overview of the literature: outcomes of stemmed revision implants after RTKA/ReRTKA

Research group	Year	All cases (PJI cases)	Metaphyseal fixation	Stem fixation	Implant type	Stem design	FU (years)	Survival	Aseptic loosening (AL)	PJI	Others
Murray et al.	1994	40 (0)		Cemented	Kinematic stabilizer (Howmedica)	*Co*	4.9	*N/A*	2.5% (RLL 32%)	*N/A*	*N/A*
Whaley et al.	2003	38 (1)		Cemented	Kinematic stabilizer (Howmedica)	*Co*	10.1	10y-, 11y-survival 96.7%, 95.7% (for AL)	5.3%	2.6%	10.5% failure
Pradhan et al.	2004	51 (23)		Cemented	ENDO Model (LINK)	*Co*	4	*N/A*	*N/A*	4.3%	*N/A*
Joshi et al.	2008	78 (0)		Cemented	ENDO Model (LINK)	*Co*	7.8	*N/A*	5.1%	2.6%	*N/A*
Schmitz et al.	2013	38 (0)	Cones	Cemented	ENDO Model (LINK) 41% rotational knee, 59% hinge knee	*Co*	3.1	*N/A*	5.3%	0%	5.3% ReRTKA
Abdelaziz et al.	2019	25 (0)	Cones	Cemented	ENDO Model (LINK) 12 hinged, 13 rotating hinged	*Co*	10.5	10y- survival 75%	20% (4/5 pure hinged)	12%	52% ReRTKA. 80% of asept failure in pure hinged and no-cones RTKA
Brown et al.	2019	100 (19)		Cemented	ENDO Model RH (LINK)	*Co*	8.2	1y-, 5y-survival 99%, 95% 5y-aseptic survival 97%	1%	5%	8% ReRTKA 29% Compli (17 infections)
Ohlmeier et al.	2021	52 (17)	Cones	Cemented	ENDO Model RH (LINK)	*Co*	1.8	*N/A*	1.9% (RLL 10.7%)	3.8%	7.7% ReOP
Bertin et al.	1985	53 (0)		Cementless	49% stemmed ICKH prosthesis 51% Freeman–Samuelson prosthesis	*Co*	1.5	*N/A*	0% (RLL 23% f, 26% t)	3.8%	35.8% Compli
Gofton et al.	2002	89 (0)		16% cemented	Coordinate (DePuy)	*Cy (Co)*	5.9	9y-survival 93%	*N/A*	*N/A*	5.6% failure
De Martino et al.	2015	18 (13)	Cones	17% cemented	33.3% LCCK, 66.7% RHK (NexGen, Zimmer)	*Cy*	6	*N/A*	0%	11%	11.1% ReRTKA
Bae et al.	2013	224 (22)		29% cemented	PFC (DePuy), 89.7% stem extension	Cy (Co)*	8.1	5y-, 8y-, 10y-survival 97.2%, 91.6%, 86.1%	2.7%	3%	8.9% ReRTKA
Villanueva-Martinez et al.	2013	21 (5)	Cones	43% cemented	47.6% RHK, 52.4% LCCK (Zimmer)	*Cy*	3	*N/A*	0%	9.5%	4.8% ReRTKA, 52.4% Compli
Gómez-Vallejo et al.	2018	67 (0)	Sleeves	43% cemented	Cemented: NK II, (Zimmer) Cementless: Sigma TC3 (DePuy)	Cy (Co)* *Cy*	7	5y-, 10y-survival 93%, 84% *(cemented)* 94%, 94% *(cementless)*	Cemented: 0% 6 possible AL (2 f, 4 t) Cementless: 0% 5 possible AL (4 f, 1 t)	4.5%	9% failures
Chalmers et al.	2017	227 (84)	Sleeves	48% cemented	22% Sigma PS, 73% TC3, 4% LPS (DePuy)	Cy (Co)*	3.2	5y-survival 96%	1.3%	5.3%	6.6% ReRTKA 11.8% Compli, 12.7%ReOP
Fehring et al.	2003	113 (33) 202 metaph. stems		53% cemented	49% Insall–Burstein II MR (Zimmer) 44% PFC Modular Revision (DePuy)	*Cy* Cy (Co)*	> 2	*N/A*	0% cemented stems 10% cementless stems (8 f, 2 t)	*N/A*	*N/A*
﻿Lachiewicz et al.	2012	27 (13)	Cones	57% cemented	81.5% LCCK (18.5% PS), 11.1% RHK, 7.4% NKII (Zimmer)	*Cy* Cy (Co)*	3.3	*N/A*	3.7%	3.7%	7.4% ReRTKA
Mabry et al.	2007	70 (0)		70% cemented	PFC 41% PS, 59% SC (DePuy)	Cy (Co)*	10.2	5y-, 10y-survival 98%, 92%	7.1% (RLL 9 f, 13 t)	2.9%	10% failure, 9% Compli
Wilke et al.	2014	234 (0)	Sleeves	98% cemented	TC3 (DePuy)	Cy (Co)*	9	5y-, 10y-survival 91%, 81%	9.4% (AL, osteolysis or pain)	7.7%	17.1% ReRTKA
Haas et al.	1995	65 (0)		Cementless	Insall-Burstein (Zimmer)	*Cy*	3.5	8y-survival 83%	3% (RLL 1% f, 7% t)	*N/A*	*N/A*
Barrack et al.	2000	14 (0)	Sleeves	Cementless	S-ROM Noiles (DePuy)	*Cy*	4.2	*N/A*	0%	0%	7% ReOP
Shannon et al.	2003	63 (21)		Cementless	46% PFC (DePuy) 54% Genesis I (Smith&Nephew)	*Cy*	5.8	*N/A*	16% (10% revised, 6.3% radiographic)	6%	19% failure
Peters et al.	2009	184 (53)		Cementless	Vanguard, 45% PS (Zimmer Biomed)	*Cy*	4.1	*N/A*	1.1% (only f)	7.1%	8.2% failure
Wood et al.	2009	135 (34)		Cementless	Genesis II Revision, 49% PS (Smith&Nephew)	*Cy*	5	Aseptic 12y-survival 98%	2% only t (RLL 19%)	2%	6% failure
Rao et al.	2013	26 (9)	Cones	Cementless	RHK (Zimmer)	*Cy*	3	*N/A*	0%	7.7%	3.8% ReRTKA
Derome et al.	2014	29 (7)	Cones	Cementless	62% LCCK, 37.8% LCCK PS (Zimmer)	*Cy*	2.8	*N/A*	3.5%	6.9%	6.9% ReRTKA, 17.2% ReOP
Huang et al.	2014	83 (20)	Sleeves	Cementless	88% Sigma PS 12% S-ROM Noiles (DePuy)	*Cy*	2.4	Survival 92.8%	3.6% (progressive RLL in 12 stems)	7.2%	16.9% ReOP
Jensen et al.	2014	36 (15)	Cones	Cementless	16.7% PS, 38.9% LCCK, 44.4% RHK (Zimmer)	*Cy*	3.9	*N/A*	5.6% (RLL 10%)	5.6%	11.1% ReRTKA
Lique et al.	2014	77		Cementless	Optetrak CCK (Exactech)	*Cy*	9	2y-, 5y-, 8y-survival 92.7%, 87.8%, 87.8%	6.6% (aseptic failure)	7.4%	29% major Compli
Bugler et al.	2015	35 (0)	Sleeves	Cementless	PFC Sigma (DePuy)	*Cy*	3.3	*N/A*	0%	0%	0% ReRTKA, 25.7% Compli
Graichen et al.	2015	121 (0)	Sleeves	Cementless	63.6% Sigma PS, 22.3% TC3, 14% S-ROM® Noiles (DePuy)	*Cy*	3.6	Aseptic sleeve-survival 98.3%	4.1% (RLL 5.8%)	3.3%	11.4% ReRTKA
Dalury et al.	2016	40 (6)	Sleeves	Cementless	15% S-ROM Noiles, 85% Sigma RKS (DePuy)	*Cy*	4.8	*N/A*	2.5%	0%	2.5% ReRTKA
Girerd et al.	2016	52 (19)	Cones	Cementless	73% RHK, 27% LCCK (Zimmer)	*Cy*	2.8	*N/A*	0%	7.7%	7.7% ReRTKA, 9.6% Compli
Martin-Hernandez et al.	2016	134	Sleeves	Cementless	Sigma TC3 (DePuy)	*Cy*	6	Aseptic survival 100%	0%	1.3%	12% Compli, 2.3% ReOP
Watters et al.	2017	116	Sleeves	Cementless	2.6% Hinge, 28.4% LCS VVC, 56% Sigma TC3, 13% Sigma PS (DePuy)	*Cy*	5.3	Sleeve survival 98.5%	0.9%	5.2%	16.4% ReOP
Agarwal et al.	2018	104	Sleeves	Cementless	Sigma TC3 (DePuy)	*Cy*	8	*N/A*	6.7%	4.8%	22.1% ReRTKA
Burastero et al.	2018	60 (60)	Cones	Cementless	30% RHK, 70% LCCK (Zimmer)	*Cy*	3.6	Survival 90%	5% (RLL 13.3%)	3.3%	10% ReOP
Fedorka et al.	2018	50 (25)	Sleeves	Cementless	Sigma TC3, LCS Complete RKS (DePuy)	*Cy*	4.9	*N/A*	4%	2.7%	6.8% ReRTKA, 18.9% ReOP
Klim et al.	2018	56 (56)	Sleeves	Cementless	80.4% LCS Complete RKS, 35.7% S-ROM Noiles (DePuy)	*Cy*	5.3	*N/A*	0%	16%	16% ReRTKA
Wirries et al.	2019	62 (17)	Sleeves	Cementless	51.1% TC3, 48.9% S-ROM Noiles (DePuy)	*Cy*	5	Survival 87.2% Aseptic survival 93.6%	6.4% (RLL 29.4%)	6.4%	12.8% failure
Algarni et al.	2020	27 (3)	Sleeves	Cementless	88.9% TC3, 3.7% S-ROM Noiles, 7.4% LPS (DePuy)	*Cy*	4.1	Survival 96.3% Aseptic survival 100%	0%	*N/A*	3.7% ReRTKA
Berti et al.	2020	56 (0)		Cementless	AMP Revision (MicroPort)	*Cy*	8.8	15y-survival 94.1%	1.7% (RLL 15.8%)	3.4%	
Bloch et al.	2020	319 (70)	Sleeves	Cementless	PFC Sigma PS, TC3, S-ROM Noiles, LPS (DePuy)	*Cy*	7.6	3y-, 5y-, 10y-survival 99.1%, 98.7%, 97.8%	0% (RLL 2.8% sleeves)	1.3%	1.6% ReRTKA
Erivan et al.	2020	53 (2)	Cones	Cementless	70.5% PS, 19.7% CCK, 9.8% Hinged (Zimmer)	*Cy*	2	5y-survival 93.4%	3.5%	7%	8.8% ReRTKA
Klim et al.	2020	93 (52)	Sleeves	Cementless	Complete RKS CCK (DePuy)	*Cy*	6.3	*N/A*	0%	15	﻿18.2% ReRTKA
Lai et al.	2020	51 (0)	Sleeves	Cementless	33.3% TC3 (DePuy) 66.7% LCCK (Zimmer)	*Cy*	2	*N/A*	*N/A*	14.7%	*N/A*
Lee et al.	2020	65 (0)		Cementless	NexGen LCCK (Zimmer)	*Cy*	> 2	*N/A*	26%	*N/A*	RF for f AL: males, bone defect RF for t AL: malalignment
Panesar et al.	2021	99 (33)		Cementless	S-ROM Noiles RHK (DePuy)	*Cy*	7	7y-survival 81% Aseptic survival 90%	2%	10%	18.2% ReRTKA, 26% Compli
Gurel et al.	2021	30 (8)	Sleeves	Cementless	Sigma TC3 (DePuy)	*Cy*	6.9	*N/A*	0%	0%	13.3% ReOP
Manopoulos et al.	2012	46 (22)	Sleeves	Cementless	Sigma TC3 (DePuy)	*Cy*	8.5	10y-survival 90%	2.2% (RLL 24%)	*N/A*	4.3% failures
von Hintze et al.	2021	125 (26)	10% cones	*N/A*	NexGen RHK (Zimmer)	*Cy*	7.3 *median*	10y-survival 82.4% *(excl. patella revisions)*	0.8%	7.2%	12% failure
Müller et al.	2008	89		*N/A*	RT-PLUS Solution (Smith&Nephew)	Cy (Co)*	6.3	1y-, 5y-survival 96.2%, 93%	0.75%	4.6%	0% failure
Gililland et al.	2014	82 (0)	12% Cones/sleeves	60% cemented	*N/A* Constraint: cemented 27%, cementless 94%	*N/A*	6–10	*N/A*	Cemented: 4% Cementless: 3–6%	Cemented: 0% Cementless: 2.4%	Failure: 6–8% (cemented), 9–10% (cementless)
Fleischman et al.	2017	223 (57)	36,8% Cones/sleeves	25% cemented	*N/A*	*N/A*	5.1	5y-, 10y- survival 96.5%, 83% *(cemented)* 95%, 77.2% *(cementless)﻿*	Cemented: 6.5% *(4.6% clinical, 1.9% radiographic)* Cementless: 4.4% *(1.9% clinical, 2.5% radiographic)*	Cemented: 7.4% Cementless: 5.7%	*N/A*
Chalmers et al.	2021	163 (46)	Cones	26% cemented	VVC (65%) or hinged systems (32%)	*N/A*	2.5	2y-survival 96%	1.3%	10%	14% ReOP
Leta et al.	2015	1016 (0)		86% cemented	Profix, NexGen, LCS, Genesis, LCS Complete, Duracon	*N/A*	4.5 *median*	5y-, 10y-, 15y-survival 85%, 78%, 71%	9% f, 17% t	28%	14.3% failure Cemented vs. cementless: no diff
Kamath et al.	2015	66 (26)	Cones	94% cemented	11% PS, 50% VVC, 38% hinged	*N/A*	5.7	Survival ﻿93.9%	3% (RLL 12%)	11%	27% Compli, 24% ReOP
Nelson et al.	2015	67 (0)		95% cemented	*N/A*	*N/A*	> 2	*N/A*	*N/A*	*N/A*	9% ReRTKA, 9% failure
Howard et al.	2011	24 (7)	Cones	Cemented	Various PS, CCK, RH systems	*N/A*	2.8	*N/A*	0%	0%	0% ReRTKA, 21% ReOP
Potter et al.	2016	157 (47)	Cones	Cemented	60% CCK, 35% Hinged, 4% PS	*N/A*	5	Survival 70%	6.4%	13.4%	28.7% ReOP
Hernandez et al.	2021	62	Cones	Cemented	3% UC, 23% PS, 66% VVC, 8% Hinged	*N/A*	7.6	8y-survival 62%	12.9% (RLL 30%)	17.7%	40.3% ReOP
Bohl et al.	2018	98 (24)	50% Cones	Cementless	*N/A*	*N/A*	3.5	*N/A*	2% (non-cone)	8.2% 10% (non-cone) 6% (cone)	Non-cone: 31% Compli, 28% ReOP Cone: 39% Compli, 32.7% ReOP
Vince and Long	1995	44		*N/A*	*N/A*	*N/A*	2–6	*N/A*	4.5%	*N/A*	*N/A*
Suarez et al.	2008	566 (123)		*N/A*	*N/A*	*N/A*	﻿3.3	12y-survival 82%	19%	46%	﻿12% failure 4.3% vs. 21% ReRTKA (after aseptic vs. septic)
Aggarwal et al.	2014	(a) age < 50: 84 (19) (b) age > 60: 84 (25)		*N/A*	*N/A*	*N/A*	5.6	6y-survival 71%^a^, 66.1%^b^	2^nd^ RTKA: 8%^a^, 13%^b^ 3-5^th^ RTKA: 38%^a^, 50%^b^	2^nd^ RTKA: 32%^a^, 50%^b^ 3-5^th^ RTKA: 63%^a^, 50%^b^	30% ReOP^a^ 31% ReOP^b^
Agarwal et al.	2019	95 (27)		*N/A*	*N/A*	*N/A*	5.2	*N/A*	30.5%	32.6%	*N/A*
Geary et al.	2020	1632 (361)		*N/A*	*N/A*	*N/A*	5.1	*N/A*	20.9%	38.5%	22.8% failure

*FU* follow-up, *AL* aseptic loosening, *PJI* periprosthetic joint infection, *TKA* total knee arthroplasty, *RTKA* revision total knee arthroplasty, *f* femoral, *t* tibial, *N/A* not available, *Co* conical, *Cy* cylindrical, *Cy-Co* cylindrical–conical combined design, *y* years, *RLL* radiolucent lines, *ReRTKA* re-revision of TKA implants, *Compli* = complications, *ReOP* re-operation

**Table 5:** Overview of the literature: outcomes of stemmed RTKA implants in primary TKA

Research group	Year	Cases (N)	Stem fixation	Implant type	Stem design	FU (years)	Survival	Asept. Loosening (AL)	PJI	Others
Engelbrecht et al.	1981	1074 TKA	Cemented	ENDO Model (LINK)	*Co*	> 10	*N/A*	6%	2%	*N/A*
Rand et al.	1987	38 (15 TKA, 23 RTKA)	Cemented	Kinematic RH (Howmedica)	*Co*	4–5	10y-survival 60%	13% (RLL 25% f, 50% t)	16%	*N/A*
Shaw et al.	1989	38 (20 TKA, 18 RTKA)	Cemented	Kinematic RH (Howmedica)	*Co*	4	*N/A*	7% TKA, 20% RTKA (RLL 45% TKA, 52% RTKA)	5% (TKA), 11% (RTKA)	*N/A*
Blauth and Hassenpflug	1990	497 TKA	Cemented	Blauth (Aesculap)	*Co*	4	10y-survival 89%	1.2%	3%	*N/A*
Böhm and Holy	1998	422 TKA	Cemented	Blauth (Aesculap)	*Co*	20	Survival 93.6%	0.7%	3.8%	*N/A*
Argenson and Aubaniac	2000	194 TKA	Cemented	ENDO Model (LINK)	*Co*	6–7	Survival > 90%	*N/A*	2.5%	6% Compli
﻿Zinck & Sellckau	2000	﻿2682 TKA	Cemented	﻿ENDO Model (LINK)	*Co*	5–6	Survival > 95%	1%	1.6%	11.4% Compli
Springer et al.	2001	69 (12 TKA, 57 RTKA)	Cemented	Kinematic RH (Howmedica)	*Co*	6	*N/A*	*N/A*	*N/A*	32% Compli
Himanen et al.	2002	25 (4 TKA, 21 RTKA)	Cemented	AGC DA monobloc (Biomet UK)	*Co*	2.3	*N/A*	0%	4%	20% Compli
Petrou et al.	2004	100 TKA	Cemented	ENDO Model (LINK)	*Co*	11	15y-survival 96.1%	0%	2%	*N/A*
Steckel et al.	2005	227 TKA	Cemented	Blauth (Aesculap)	*Co*	10	10y-survival 90%	1.3% (only t)	3.5%	*N/A*
Pour et al.	2007	44 (17 TKA, 27 RTKA)	93% Cemented 7% Cementless	70.5% Kinematic RH (Howmedica) 29.5% Finn RH (Biomed)	*Co* *Cy(f)* *Cy-Co(t)*	4.2	1y-, 5y-survival 79.6%, 68.2%	9.1%	6.8%	*N/A*
Deehan et al.	2008	72 (15 TKA, 57 RTKA)	Cemented	Kinematic RH (Howmedica)	*Co*	10	5y-survival 90%	1.4%	5.6%	*N/A*
Bae et al.	2009	11 TKA *Charcot knees*	Cemented	﻿ENDO Model (LINK)	*Co*	12.3	10y-survival 72.7%	0%	9.1%	27.3% Compli
Guenoun et al.	2009	85 (*52* TKA, 33 RTKA)	Cemented	﻿ENDO Model (LINK)	*Co*	3	3y-survival 89.4%	3.5%	10.6%	28.2% Compli
Efe et al.	2012	49 (21 TKA, 28 RTKA)	Cemented	ENDO Model (LINK)	*Co*	4.7	Survival 95% TKA, 76% RTKA	0% (TKA), 7.1% (RTKA)	4.8% (TKA), 32% (RTKA)	Failure: 14.3% (TKA), 53.6% (RTKA)
Yang et al.	2012	50 TKA	Cemented	ENDO Model (LINK)	*Co*	15	10y-survival 87%	0% (RLL 10%)	14%	14% failure
Bistolfi et al.	2013	72 TKA	Cemented	ENDO Model RH (LINK)	*Co*	14.5	1y-, 5y-, 10y-, 15y-survival 88.7%, 86%, 79.8%, 75.8%	4.1%	8.3%	25% failure
Gehrke et al.	2014	141 TKA	Cemented	ENDO Model (LINK)	*Co*	13.5	Survival 90%	0.5% (f)	2%	8% failure
Hernandez-Vaquero et al.	2010	26 (5 TKA, 21 RTKA)	Cemented (100% t, 54% f)	Modular RH Prosthesis (Stryker)	*Cy-Co*	3.8	*N/A*	0% (RLL 19%)	7.7%	11.5% ReOP
Kowalczewski et al.	2014	12 TKA	Cementless	Modular RH Prosthesis (Stryker)	*Cy-Co*	10–12	10y-survival 100%	0% (RLL 25% t)	0%	0% failures
Westrich et al.	2000	24 (9 TKA, 15 RTKA)	Cemented	Finn RH (Biomet)	*Cy(f)* *Cy-Co(t)*	2–3	survival 100%	0%	*N/A*	*N/A*
Barrack et al.	2001	23 (2 TKA, 21 RTKA)	Cementless	S-ROM Noiles (DePuy)	*Cy*	4.8	*N/A*	0%	*N/A*	*N/A*
Kowalczewski et al.	2004	28 (15 TKA, 13 RTKA)	*N/A*	AGC DA (Biomet UK)	*Cy*	0.5	*N/A*	1%	0%	2 Compli
Springer et al.	2004	26 (4 TKA, 22 RKTA)	Cemented	Modular Segmental Kinematic RH (Howmedica)	*Cy*	5	10y-survival < 70%	15.4% (RLL 42.3%)	19.2%	31% Compli, 27% ReOP
Brinsuk	2009	155 (142 TKA, 13 RTKA)	Cemented	RT-PLUS Solution (Smith&Nephew)	Cy (Co)*	2		0.6%	4%	13.5% Compli
Maynard et al.	2014	127 TKA	Cementless	NexGen LCCK (Zimmer)	*Cy*	9.2	10y-survival 97.6%	0%	1.6%	19.7% Compli, 10.2% RTKA
Cholewinski et al.	2015	43 TKA	Cementless	NexGen LCCK (Zimmer)	*Cy*	12.7	11y-survival 88.5%, aseptic 97.7%	0%	4.7%	16% Compli, 7% RTKA
Lique et al.	2015	89 TKA	Cementless	Optetrak CCK (Exactech)	*Cy*	9	2y-, 8y-survival 93.8%, 90.1%	2.2%	6.7%	7.8% failure
Feng et al.	2016	48 TKA	Cementless	NexGen LCCK (Zimmer)	*Cy*	6	6y-survival 97.9%	2.1% t, (RLL 8.3%)	0%	*N/A*
Ye et al.	2016	47 (31 TKA, 16 RTKA)	Cementless	NexGen LCCK (Zimmer)	*Cy*	5.5	*N/A*	0% (RLL 4%)	3.2% (TKA), 6.3% (RTKA)	*N/A*
Johnson et al.	2019	21 TKA (age < 60)	Cemented	Sigma TC3 (DePuy)	Cy (Co)*	5.5	Survival 100%	0% (RLL 52%)	0%	
Panda et al.	2019	79 (20 TKA, 59 RTKA) & cones	Cementless	67% LCCK, 33% RHK (Zimmer)	*Cy*	6.6	Survival 95%	0% (RLL 8.9%)	2.5%	26.6% Compli, 5% ReOP
Gill et al.	2020	43 (12 TKA 31 RTKA & sleeves)	Cementless	32.6% Sigma PS, 44.2% Sigma TC3, 23.3% S-ROM Noiles (DePuy)	*Cy*	5.4	*N/A*	0% (RLL 4.6%)	2.3%	0% ReRTKA
Mancino et al.	2020	49 TKA	93.4% (short) cemented 6.4% (long) cementless	NexGen LCCK (Zimmer)	*Cy*	9	survival 93.6% (100% for AL)	0%	4.3%	*N/A*
Backer et al.	2012	964 TKA	*N/A*	various RH prostheses	*N/A*	5	5y-survival 96.98%	0.3%	0.8%	2% RTKA
Martin et al.	2016	28.667 TKA	*N/A*	427 CCK, 246 RH (DePuy, Zimmer, Stryker)	*N/A*	10.1	10y-survival 90% (CCK) and 74.6% (RH) 20y-survival 72.8% (CCK) & 40.3% (RH)	4.4% (CCK), 6.1% (RH)	1.2% (CCK), 3.7% (RH)	RTKA: 7.3% (CCK), 17.1% (RH)
Tripathi et al.	2016	100 TKA (BMI > 30)	*N/A*	CCK prostheses	*N/A*	7	*N/A*	1%	1%	*N/A*
Siqueira et al.	2017	685 (247 TKA, 315 aseptic RTKA, 123 septic RTKA)	82% Cemented	various RH prostheses	*N/A*	8.2	TKA: 10y-survival 88.5% RKTA: 10y-survival 75.8% (asept) 54.6% (sept)	TKA: 0.8% RTKA: 1.9%	TKA: 5.3% RTKA: 22.8%	*N/A*

*FU* follow up, *AL* aseptic loosening, *PJI* periprosthetic joint infection, *TKA* total knee arthroplasty, *RTKA* revision total knee arthroplasty, *f* femoral, *t* tibial, *N/A* not available, *Co* conical, *Cy* cylindrical, *Cy-Co* cylindrical-conical combined designs, *y* years, *RLL* radiolucent lines, *ReRTKA* re-revision of TKA implants, *Compli* complications, *ReOP* re-operation

In the solely RTKA publication group (1985–2021), 8623 cases (involving 971 infections) were included. In 41 of 67 (61.2%) studies, a metaphyseal fixation (cones and sleeves) was used in variable frequencies, mostly combined with cementless cylindrical stems. An overall of 33 ± 42% of stems were cemented (Co 100% vs. Cy 11.6%). The mean FU time in years was 5.2 ± 7.7 (1.5–10.5). Comparing the data selected between Co vs. Cy stems after excluding studies with undefined implants, no differences in overall outcomes could be detected. With similar 10-year survival rates of 85.9 ± 15.3% (75–96.7%) for Co and 88.9 ± 5.6% (81–97.8%) for Cy, the incidences of aseptic loosening and PJI were identical for both, Co: 5.1 ± 6.3% (0–20%) vs. Cy: 3.5 ± 5% (0–26%) and Co: 4.3 ± 3.5% (0–12%) vs. Cy: 5.7 ± 4.1% (0–16%), respectively. Equal relations of outcomes were observed also in the comparison of the RTKA implants in primary ± revision group (Fig. 4a, b).

Comparing the literature data presented with our findings, a lower overall incidence of aseptic loosening and PJI was obviously noticeable. This can be attributed to a cohort inhomogeneity (TKA vs. RTKA, risk factors and complexity of cases, number or previous revisions, septic vs. aseptic, use of metaphyseal fixation, FU time, implant design and fixation technique, missing values, study design, industry influence, etc.) that leads to a wide discrepancy of outcomes that range from excellent [36, 40, 55, 68–77] to moderate results [16, 20, 24, 41, 62, 78–80]. However, when considering recent (2008–2020) high-volume studies that are not associated with a certain implant, the incidence rates of aseptic loosening and PJI are more representative and coincide with our data: Suarez et al. in a series of 566 RTKA cases (including 123 PJI) published a 12-year survival of 82% with 19% aseptic loosening and 46% PJI [19]. Aggarwal et al. followed 168 RTKA cases (44 PJI) for a mean time of 5.6 years. Complex cases with ≥ 3 previous revisions were associated with increased risk for aseptic loosening and PJI (38–50% and 50–63%, respectively). In a current single-center review of 1632 RKTA (361 PJI) and a mean FU of 5.1 years, the RTKA failure rates due to aseptic loosening was 21% and due to PJI 38.5% [24].

When assessing studies using Co vs. Cy stem designs, the majority of published data on Cy stems are performed in press-fit/hybrid fixation techniques, whereas Co stems are commonly full cemented, which corresponds to the common use in daily praxis. However, there are cases where cylindrical stems can be cemented (e.g., poor bone quality, metaphyseal stems or stems with < 4 cm diaphyseal engagement, bone deformities, implant availability) [23, 49, 81, 82] (Table 4). Fehring et al. compared cemented (53%) with cementless metaphyseal stems on 113 RTKA (33 PJI) and found in an FU > 2 years 0% vs. 10% aseptic loosening in favor of cemented stems [54]. Gililland et al. followed with a multi-center study comparing cemented stems with diaphyseal engaging press-fit stems on 82 aseptic RTKA cases. After an FU of 6–10 years, they found no differences in aseptic loosening (4% vs. 3–6%) or PJI (0% vs. 2.4%) for cemented and cementless diaphyseal stems, respectively. Re-revision rates and radiographic failure rates were similar between groups [67].

In a current study on 65 aseptic RTKA cases with cementless Cy stems, 26% of revision implants failed after 2 years FU due to aseptic loosening [62].

A study from 2004 on 26 modular segmental rotating hinge RTKA implants with cemented Cy stems (4 primary, 22 revisions) in nonneoplastic limp salvage cases and a mean FU of 5 years found 10-year survival rates < 70%, aseptic loosening in 15.4% and PJI in 19.2% with 27% reoperations and 31% complication rates [79].

In 2015, a Norwegian arthroplasty register study on 1016 cases of various RTKA implants that mostly had Cy stems (85% cemented), published at a median FU of 4.5 years PJI rates of 28%. Tibial loosening occurred in 17% vs. 9% on the femoral side. Partial component exchange, young ages and male patients were found to pose a higher risk for ReRTKA [15].

Abdelaziz et al. compared the 10-years outcomes of 25 aseptic RTKA between pure and rotating hinged designs both with cemented conical stems and 32 cones. He found aseptic loosening in 20%, from which four out of five revised RTKA implants were pure hinged. They concluded that pure hinged RKTA without cones tend toward higher loosening rates [83]. Other studies on RTKA implants with cemented Co stems published aseptic loosening rates of 7.1% for revisions [80] and 0–0.7% for primary implantations [55, 69, 70].

Despite all efforts made so far to improve outcomes by advancing implant design properties, operative techniques and the perioperative management, the RTKA failure rates are still high [14–25]. Causes of RTKA failure include PJI (21–63%), followed by aseptic loosening (20–50%), instability (3–26%), stiffness (10.5–23%), implant malposition (3%), painful knee (7.3%), extensor mechanism problems (5.2–12.8%) and periprosthetic fractures (6%) [14, 15, 19, 20, 24, 25].

Thus, highlighted by the need to improve the RTKA results, a meticulous and continuous epidemiological analysis of both patient- and surgery/implant-associated potential risks is of particular importance.

However, confronted with the fact that the existing literature is burdened with a cohort inhomogeneity and a lack of values about the exact design and proportion of conical profile of the stems used, it is difficult to examine the impact of stem conicity on the longevity of the implants and draw reliable conclusions.

When looking into the published data of hip arthroplasty, tapered stems appear to provide superior outcomes than cylindrical stems even in cases of greater bony deficiencies. In a recent multicenter analysis of 105 femoral revisions with Paprosky III–IV, Bedair et al. found at 5 years FU that modular revision stems with conical geometry were associated with lower rates of failed implant osseointegration (1.6% vs. 15.9%, *P* < 0.01) and stem re-revision (4.9% vs. 22.7%, *P* = 0.013) than cylindrical stems [29]. Despite the fact that these results may obtain some similarities with our observations in terms of impact of stem conicity on implant survivorships, they cannot be directly compared, as there are substantial differences in biomechanics and anchoring principles between the knee and hip.

Thus, this is the first observational study to provide evidence about the effect of stem conicity on the failure pattern of RTKA.

Compared to cylindrical designs, conical stems seem to have some mechanical advantages: while during press-fit fixations a longer intramedullary engagement length occurs, which might lead to reduced areas of micromotions between stem and cortical bone induced due to the differences in elasticity and rigidity modulus of the materials (less stress shielding, less peak stresses at the tip of the stem), in cases of cemented fixation a cone-shaped cement coat may theoretically reduce the shear stresses and thus the micromotions in the cement–implant interface which in the long-term could otherwise trigger implant migration and bond failure. However, there is still lack of clinical and biomechanical evidence that could confirm the above statements. Conical stems can also be removed more easily than cylindrical stems due to the short displacement required to achieve complete implant detachment [27, 28].

There are several limitations in the current study that may pose potential biases: (1) the small sample size of the subgroups and the retrospective setup of this work, (2) as aforementioned, we did not differentiate hinge designs and level of constraint and did not consider other stem design properties such as length, diameter, offset, bowed profile, and surface roughness values, which might have had also an impact on the failure pattern, (3) we did not report any follow-up time, as focus of this investigation was rather the epidemiological analysis of the failure patterns and not the evaluation of implant survivorship, (4) all values of stem profile of the RTKA stems included in the study were based on measurements conducted using the preoperative planning software MediCAD^®^, which might involve some variations and thus potential discrepancies between our measurements and the manufacturer’s values. Therefore, the validity of these results is limited. Nonetheless, it is still consistent with the available evidence. Further studies of large series including survival rates are currently being conducted by our research group to bring more clarity to this topic.

## Conclusion

PJI remains the most common reason for ReRTKA, independent of the conical profile of RTKA stem extensions. Cemented cylindrical stems may pose a greater risk for aseptic loosening than conical stem designs.
